# Dibromido(2,3-di-2-pyridyl­pyrazine-κ^2^ *N* ^2^,*N* ^3^)platinum(II)

**DOI:** 10.1107/S160053681103412X

**Published:** 2011-08-27

**Authors:** Kwang Ha

**Affiliations:** a School of Applied Chemical Engineering, The Research Institute of Catalysis, Chonnam National University, Gwangju 500-757, Republic of Korea

## Abstract

The Pt^II^ ion in the title complex, [PtBr_2_(C_14_H_10_N_4_)], has a slightly distorted square-planar environment defined by the two pyridyl N atoms of the chelating 2,3-di-2-pyridyl­pyrazine ligand and two bromide anions. In the crystal, the pyridyl rings are considerably inclined to the least-squares plane of the PtBr_2_N_2_ unit [maximum deviation = 0.064 (2) Å] with dihedral angles of 65.2 (2) and 66.0 (2)°. The nearly planar pyrazine ring [maximum deviation = 0.020 (5) Å] is almost perpendicular to the unit plane with a dihedral angle of 89.2 (2)°. Two independent weak inter­molecular C—H⋯Br hydrogen bonds, both involving the same Br atom as a hydrogen-bond acceptor, give rise to chains running along the *a* and *b* axes, forming a layer structure extending parallel to (001). The complexes are stacked in columns along the *a* axis. When viewed down the *b* axis, the successive complexes stack in the opposite direction.

## Related literature

For an isomer of the title complex, see: Ha (2011 ▶). For crystal structures of the related Pt^II^ complexes, see: Granifo *et al.* (2000 ▶); Cai *et al.* (2009 ▶).
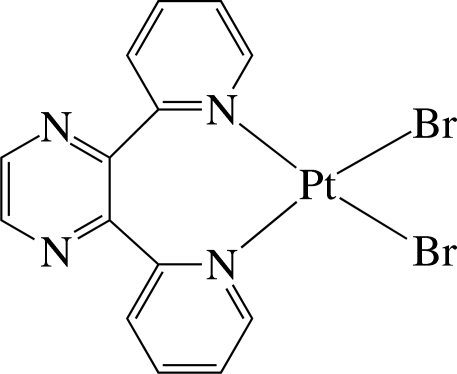


## Experimental

### 

#### Crystal data

[PtBr_2_(C_14_H_10_N_4_)]
*M* *_r_* = 589.17
Monoclinic, 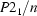

*a* = 8.4989 (12) Å
*b* = 15.348 (2) Å
*c* = 12.0277 (16) Å
β = 101.403 (3)°
*V* = 1538.0 (4) Å^3^
*Z* = 4
Mo *K*α radiation
μ = 14.32 mm^−1^
*T* = 200 K
0.18 × 0.18 × 0.13 mm

#### Data collection

Bruker SMART 1000 CCD diffractometer
Absorption correction: multi-scan (*SADABS*; Bruker, 2000 ▶) *T*_min_ = 0.105, *T*_max_ = 0.156
10118 measured reflections
3323 independent reflections
2484 reflections with *I* > 2σ(*I*)
*R*_int_ = 0.046

#### Refinement

*R*[*F* ^2^ > 2σ(*F* ^2^)] = 0.032
*wR*(*F* ^2^) = 0.067
*S* = 0.99
3323 reflections
190 parameters
H-atom parameters constrained
Δρ_max_ = 1.66 e Å^−3^
Δρ_min_ = −0.84 e Å^−3^

### 

Data collection: *SMART* (Bruker, 2000 ▶); cell refinement: *SAINT* (Bruker, 2000 ▶); data reduction: *SAINT*; program(s) used to solve structure: *SHELXS97* (Sheldrick, 2008 ▶); program(s) used to refine structure: *SHELXL97* (Sheldrick, 2008 ▶); molecular graphics: *ORTEP-3* (Farrugia, 1997 ▶) and *PLATON* (Spek, 2009 ▶); software used to prepare material for publication: *SHELXL97*.

## Supplementary Material

Crystal structure: contains datablock(s) global, I. DOI: 10.1107/S160053681103412X/xu5295sup1.cif

Structure factors: contains datablock(s) I. DOI: 10.1107/S160053681103412X/xu5295Isup2.hkl

Additional supplementary materials:  crystallographic information; 3D view; checkCIF report

## Figures and Tables

**Table 1:** Selected bond lengths (Å)

Pt1—N3	2.026 (5)
Pt1—N4	2.029 (5)
Pt1—Br1	2.4202 (8)
Pt1—Br2	2.4335 (8)

**Table 2:** Hydrogen-bond geometry (Å, °)

*D*—H⋯*A*	*D*—H	H⋯*A*	*D*⋯*A*	*D*—H⋯*A*
C6—H6⋯Br1^i^	0.95	2.88	3.524 (6)	126
C11—H11⋯Br1^ii^	0.95	2.88	3.688 (7)	143

Symmetry codes: (i) 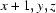; (ii) 
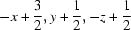
.

**Fig. 1.**
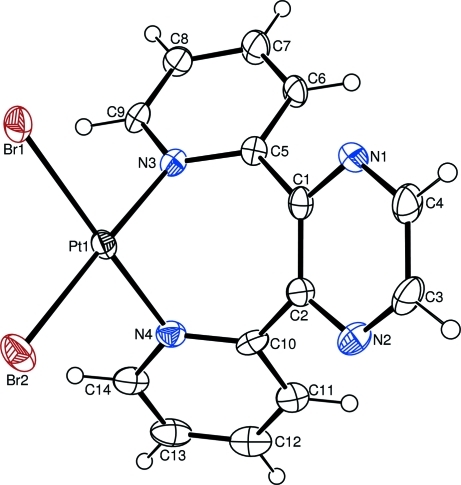
The structure of the title complex, with displacement ellipsoids drawn at the 50% probability level; H atoms are shown as small circles of arbitrary radius.

**Fig. 2.**
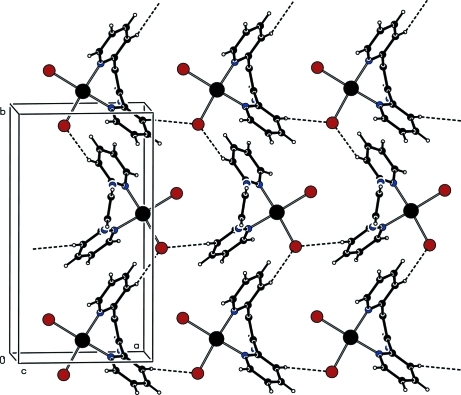
View of the hydrogen-bond interactions of the title complex. Hydrogen-bonds are drawn with dashed lines.

## supplementary crystallographic information

### Comment

The title complex, [PtBr_2_(dpp)] (dpp is 2,3-di-2-pyridylpyrazine, C_14_H_10_N_4_), is a structural isomer of the previously reported Pt(II) complex (Ha, 2011). The Pt^II^ ion has a slightly distorted square-planar environment defined by the two pyridyl N atoms of the chelating dpp ligand and two bromide anions (Fig. 1). The coordination mode of the dpp ligand is similar to that found in the mononuclear Pt(II) complexes [PtCl_2_(dpq)] (dpq = 2,3-di-2-pyridylquinoxaline) (Granifo *et al.*, 2000) and [PtCl_2_(dcdpp)] (dcdpp = 2,3-dicyano-5,6-di-2-pyridylpyrazine) (Cai *et al.*, 2009).

The N3—Pt1—N4 chelate angle of 87.7 (2)° and Br—Br repelling contribute the distortion of square, and therefore the *trans* axes are slightly bent [<Br1—Pt1—N4 = 174.08 (15)° and <Br2—Pt1—N3 = 178.19 (14)°]. The Pt—N and Pt—Br bond lengths are nearly equivalent, respectively (Table 1). In the crystal, the two pyridyl rings are considerably inclined to the least-squares plane of the PtBr_2_N_2_ unit [maximum deviation = 0.064 (2) Å] with dihedral angles of 65.2 (2)° and 66.0 (2)°, respectively. The nearly planar pyrazine ring [maximum deviation = 0.020 (5) Å] is almost perpendicular to the unit plane with a dihedral angle of 89.2 (2)°. The dihedral angle between the two pyridyl rings is 80.5 (2)°. Two independent intermolecular C—H···Br hydrogen bonds, both involving the same Br atom as an H-bond acceptor, give rise to chains running along the a and b axes, forming a layer structure extending parallel to the *ab* plane (Fig. 2 and Table 2). The complexes are stacked in columns along the *a* axis. When viewed down the *b* axis, the successive complexes stack in the opposite direction. In the columns, numerous inter- and intramolecular π-π interactions between the six-membered rings are present, the shortest ring centroid-centroid distance being 3.833 (4) Å.

### Experimental

The title complex was obtained as a byproduct from the reaction of K_2_PtBr_4_ (0.2967 g, 0.500 mmol) with 2,3-di-2-pyridylpyrazine (0.1173 g, 0.501 mmol) in H_2_O (20 ml). After stirring of the reaction mixture for 3 h at room temperature, the formed precipitate was separated by filtration, washed with H_2_O and acetone, to give the main product as a red-brown powder (0.1326 g) (Ha, 2011). The yellow byproduct (0.0299 g) was obtained from the mixture of filtrate and washing solution. Crystals suitable for X-ray analysis were obtained by slow evaporation from a CH_3_NO_2_ solution of the byproduct.

### Refinement

H atoms were positioned geometrically and allowed to ride on their respective parent atoms [C—H = 0.95 Å and *U*_iso_(H) = 1.2*U*_eq_(C)]. The highest peak (1.66 e Å^-3^) and the deepest hole (-0.84 e Å^-3^) in the difference Fourier map are located 1.95 Å and 0.89 Å from the atoms H12 and Pt1, respectively.

### Crystal data

**Table:**

[PtBr_2_(C_14_H_10_N_4_)]	*F*(000) = 1080
*M**_r_* = 589.17	*D*_x_ = 2.544 Mg m^−^^3^
Monoclinic, *P*2_1_/*n*	Mo *K*α radiation, λ = 0.71073 Å
Hall symbol: -P 2yn	Cell parameters from 4242 reflections
*a* = 8.4989 (12) Å	θ = 2.7–27.0°
*b* = 15.348 (2) Å	µ = 14.32 mm^−^^1^
*c* = 12.0277 (16) Å	*T* = 200 K
β = 101.403 (3)°	Block, yellow
*V* = 1538.0 (4) Å^3^	0.18 × 0.18 × 0.13 mm
*Z* = 4	

### Data collection

**Table:**

Bruker SMART 1000 CCD diffractometer	3323 independent reflections
Radiation source: fine-focus sealed tube	2484 reflections with *I* > 2σ(*I*)
graphite	*R*_int_ = 0.046
φ and ω scans	θ_max_ = 27.0°, θ_min_ = 2.2°
Absorption correction: multi-scan (*SADABS*; Bruker, 2000)	*h* = −9→10
*T*_min_ = 0.105, *T*_max_ = 0.156	*k* = −11→19
10118 measured reflections	*l* = −13→15

### Refinement

**Table:**

Refinement on *F*^2^	Primary atom site location: structure-invariant direct methods
Least-squares matrix: full	Secondary atom site location: difference Fourier map
*R*[*F*^2^ > 2σ(*F*^2^)] = 0.032	Hydrogen site location: inferred from neighbouring sites
*wR*(*F*^2^) = 0.067	H-atom parameters constrained
*S* = 0.99	*w* = 1/[σ^2^(*F*_o_^2^) + (0.0237*P*)^2^] where *P* = (*F*_o_^2^ + 2*F*_c_^2^)/3
3323 reflections	(Δ/σ)_max_ < 0.001
190 parameters	Δρ_max_ = 1.66 e Å^−^^3^
0 restraints	Δρ_min_ = −0.84 e Å^−^^3^

### Special details

**Table:**

Geometry. All e.s.d.'s (except the e.s.d. in the dihedral angle between two l.s. planes) are estimated using the full covariance matrix. The cell e.s.d.'s are taken into account individually in the estimation of e.s.d.'s in distances, angles and torsion angles; correlations between e.s.d.'s in cell parameters are only used when they are defined by crystal symmetry. An approximate (isotropic) treatment of cell e.s.d.'s is used for estimating e.s.d.'s involving l.s. planes.
Refinement. Refinement of *F*^2^ against ALL reflections. The weighted *R*-factor *wR* and goodness of fit *S* are based on *F*^2^, conventional *R*-factors *R* are based on *F*, with *F* set to zero for negative *F*^2^. The threshold expression of *F*^2^ > σ(*F*^2^) is used only for calculating *R*-factors(gt) *etc*. and is not relevant to the choice of reflections for refinement. *R*-factors based on *F*^2^ are statistically about twice as large as those based on *F*, and *R*- factors based on ALL data will be even larger.

### Fractional atomic coordinates and isotropic or equivalent isotropic displacement parameters (Å^2^)

**Table:**

	*x*	*y*	*z*	*U*_iso_*/*U*_eq_	
Pt1	0.52128 (3)	0.060963 (16)	0.31708 (2)	0.02517 (9)	
Br1	0.38241 (9)	−0.07734 (4)	0.30704 (6)	0.03471 (18)	
Br2	0.27287 (9)	0.14353 (5)	0.26735 (6)	0.0413 (2)	
N1	0.8219 (7)	0.0077 (3)	0.0833 (5)	0.0314 (14)	
N2	0.7631 (7)	0.1861 (4)	0.0810 (5)	0.0368 (15)	
N3	0.7311 (6)	−0.0055 (3)	0.3550 (4)	0.0238 (12)	
N4	0.6520 (7)	0.1724 (3)	0.3406 (4)	0.0284 (13)	
C1	0.8029 (8)	0.0499 (4)	0.1780 (5)	0.0265 (15)	
C2	0.7701 (8)	0.1400 (4)	0.1755 (6)	0.0304 (16)	
C3	0.7799 (10)	0.1437 (5)	−0.0117 (6)	0.042 (2)	
H3	0.7738	0.1755	−0.0802	0.051*	
C4	0.8060 (9)	0.0552 (5)	−0.0126 (6)	0.0394 (19)	
H4	0.8128	0.0272	−0.0819	0.047*	
C5	0.8339 (8)	−0.0057 (4)	0.2819 (5)	0.0231 (14)	
C6	0.9675 (8)	−0.0587 (4)	0.3011 (6)	0.0305 (16)	
H6	1.0386	−0.0590	0.2492	0.037*	
C7	0.9973 (9)	−0.1111 (5)	0.3954 (6)	0.0392 (18)	
H7	1.0881	−0.1485	0.4087	0.047*	
C8	0.8944 (8)	−0.1088 (4)	0.4708 (6)	0.0333 (17)	
H8	0.9149	−0.1436	0.5375	0.040*	
C9	0.7622 (8)	−0.0557 (4)	0.4483 (5)	0.0266 (15)	
H9	0.6909	−0.0544	0.4999	0.032*	
C10	0.7583 (9)	0.1933 (4)	0.2760 (5)	0.0307 (16)	
C11	0.8525 (9)	0.2669 (4)	0.3000 (6)	0.0397 (19)	
H11	0.9274	0.2815	0.2540	0.048*	
C12	0.8379 (10)	0.3187 (5)	0.3903 (6)	0.043 (2)	
H12	0.9026	0.3693	0.4073	0.052*	
C13	0.7293 (10)	0.2969 (5)	0.4557 (6)	0.044 (2)	
H13	0.7186	0.3322	0.5187	0.053*	
C14	0.6361 (9)	0.2243 (4)	0.4303 (5)	0.0352 (18)	
H14	0.5598	0.2096	0.4753	0.042*	

### Atomic displacement parameters (Å^2^)

**Table:**

	*U*^11^	*U*^22^	*U*^33^	*U*^12^	*U*^13^	*U*^23^
Pt1	0.02370 (16)	0.02954 (16)	0.02362 (14)	0.00465 (12)	0.00797 (11)	0.00200 (11)
Br1	0.0235 (4)	0.0404 (4)	0.0414 (4)	−0.0026 (3)	0.0092 (3)	−0.0002 (3)
Br2	0.0357 (5)	0.0529 (5)	0.0367 (4)	0.0204 (4)	0.0108 (3)	0.0100 (3)
N1	0.035 (4)	0.029 (3)	0.033 (3)	0.004 (3)	0.014 (3)	−0.002 (3)
N2	0.043 (4)	0.035 (4)	0.033 (3)	0.001 (3)	0.009 (3)	0.012 (3)
N3	0.026 (3)	0.024 (3)	0.021 (3)	−0.004 (2)	0.003 (2)	0.000 (2)
N4	0.032 (4)	0.025 (3)	0.028 (3)	0.004 (3)	0.005 (3)	0.003 (2)
C1	0.019 (4)	0.036 (4)	0.025 (4)	−0.003 (3)	0.005 (3)	0.001 (3)
C2	0.023 (4)	0.028 (4)	0.042 (4)	−0.001 (3)	0.009 (3)	0.004 (3)
C3	0.058 (6)	0.041 (5)	0.033 (4)	−0.002 (4)	0.021 (4)	0.013 (3)
C4	0.042 (5)	0.051 (5)	0.029 (4)	0.006 (4)	0.014 (3)	0.014 (3)
C5	0.023 (4)	0.027 (4)	0.018 (3)	−0.002 (3)	0.001 (3)	0.000 (3)
C6	0.017 (4)	0.035 (4)	0.042 (4)	0.003 (3)	0.012 (3)	0.006 (3)
C7	0.024 (4)	0.044 (5)	0.050 (5)	0.008 (3)	0.010 (4)	0.013 (4)
C8	0.030 (4)	0.031 (4)	0.038 (4)	0.004 (3)	0.005 (3)	0.006 (3)
C9	0.025 (4)	0.031 (4)	0.024 (3)	−0.006 (3)	0.004 (3)	0.004 (3)
C10	0.035 (4)	0.022 (4)	0.032 (4)	−0.001 (3)	0.001 (3)	0.008 (3)
C11	0.042 (5)	0.032 (4)	0.046 (5)	0.001 (4)	0.012 (4)	0.003 (4)
C12	0.045 (5)	0.029 (4)	0.048 (5)	0.000 (4)	−0.009 (4)	−0.004 (4)
C13	0.067 (6)	0.030 (5)	0.031 (4)	0.009 (4)	0.002 (4)	−0.005 (3)
C14	0.055 (5)	0.030 (4)	0.019 (4)	0.009 (4)	0.007 (3)	0.002 (3)

### Geometric parameters (Å, °)

**Table:**

Pt1—N3	2.026 (5)	C4—H4	0.9500
Pt1—N4	2.029 (5)	C5—C6	1.380 (8)
Pt1—Br1	2.4202 (8)	C6—C7	1.373 (9)
Pt1—Br2	2.4335 (8)	C6—H6	0.9500
N1—C1	1.347 (8)	C7—C8	1.379 (9)
N1—C4	1.349 (8)	C7—H7	0.9500
N2—C3	1.323 (8)	C8—C9	1.371 (9)
N2—C2	1.329 (8)	C8—H8	0.9500
N3—C9	1.344 (7)	C9—H9	0.9500
N3—C5	1.357 (7)	C10—C11	1.381 (9)
N4—C10	1.341 (8)	C11—C12	1.372 (9)
N4—C14	1.368 (8)	C11—H11	0.9500
C1—C2	1.410 (9)	C12—C13	1.367 (10)
C1—C5	1.493 (8)	C12—H12	0.9500
C2—C10	1.479 (9)	C13—C14	1.366 (10)
C3—C4	1.376 (9)	C13—H13	0.9500
C3—H3	0.9500	C14—H14	0.9500
			
N3—Pt1—N4	87.7 (2)	C6—C5—C1	118.7 (6)
N3—Pt1—Br1	88.20 (14)	C7—C6—C5	119.7 (6)
N4—Pt1—Br1	174.08 (15)	C7—C6—H6	120.2
N3—Pt1—Br2	178.19 (14)	C5—C6—H6	120.2
N4—Pt1—Br2	91.13 (15)	C6—C7—C8	119.5 (7)
Br1—Pt1—Br2	93.08 (3)	C6—C7—H7	120.3
C1—N1—C4	117.0 (6)	C8—C7—H7	120.3
C3—N2—C2	117.7 (6)	C9—C8—C7	119.1 (6)
C9—N3—C5	119.6 (5)	C9—C8—H8	120.5
C9—N3—Pt1	119.7 (4)	C7—C8—H8	120.5
C5—N3—Pt1	120.4 (4)	N3—C9—C8	121.7 (6)
C10—N4—C14	120.0 (6)	N3—C9—H9	119.2
C10—N4—Pt1	122.2 (4)	C8—C9—H9	119.2
C14—N4—Pt1	117.6 (5)	N4—C10—C11	120.2 (6)
N1—C1—C2	120.7 (6)	N4—C10—C2	120.3 (6)
N1—C1—C5	113.7 (6)	C11—C10—C2	119.5 (6)
C2—C1—C5	125.4 (6)	C12—C11—C10	120.0 (7)
N2—C2—C1	121.0 (6)	C12—C11—H11	120.0
N2—C2—C10	113.9 (6)	C10—C11—H11	120.0
C1—C2—C10	124.7 (6)	C13—C12—C11	119.3 (7)
N2—C3—C4	122.4 (6)	C13—C12—H12	120.3
N2—C3—H3	118.8	C11—C12—H12	120.3
C4—C3—H3	118.8	C14—C13—C12	119.9 (7)
N1—C4—C3	121.1 (7)	C14—C13—H13	120.0
N1—C4—H4	119.4	C12—C13—H13	120.0
C3—C4—H4	119.4	C13—C14—N4	120.5 (7)
N3—C5—C6	120.5 (6)	C13—C14—H14	119.8
N3—C5—C1	120.8 (6)	N4—C14—H14	119.8
			
N4—Pt1—N3—C9	115.6 (5)	N1—C1—C5—C6	45.0 (8)
Br1—Pt1—N3—C9	−60.1 (4)	C2—C1—C5—C6	−129.7 (7)
N4—Pt1—N3—C5	−70.2 (5)	N3—C5—C6—C7	−0.6 (10)
Br1—Pt1—N3—C5	114.1 (4)	C1—C5—C6—C7	−179.3 (6)
N3—Pt1—N4—C10	62.4 (5)	C5—C6—C7—C8	−1.1 (11)
Br2—Pt1—N4—C10	−116.2 (5)	C6—C7—C8—C9	1.6 (11)
N3—Pt1—N4—C14	−113.5 (5)	C5—N3—C9—C8	−1.2 (9)
Br2—Pt1—N4—C14	67.8 (5)	Pt1—N3—C9—C8	173.0 (5)
C4—N1—C1—C2	−0.9 (9)	C7—C8—C9—N3	−0.4 (10)
C4—N1—C1—C5	−175.9 (6)	C14—N4—C10—C11	0.3 (10)
C3—N2—C2—C1	3.3 (10)	Pt1—N4—C10—C11	−175.6 (5)
C3—N2—C2—C10	177.1 (6)	C14—N4—C10—C2	−176.9 (6)
N1—C1—C2—N2	−2.5 (10)	Pt1—N4—C10—C2	7.3 (9)
C5—C1—C2—N2	171.9 (6)	N2—C2—C10—N4	128.9 (7)
N1—C1—C2—C10	−175.6 (6)	C1—C2—C10—N4	−57.6 (10)
C5—C1—C2—C10	−1.2 (11)	N2—C2—C10—C11	−48.3 (9)
C2—N2—C3—C4	−0.8 (11)	C1—C2—C10—C11	125.3 (7)
C1—N1—C4—C3	3.3 (10)	N4—C10—C11—C12	0.2 (11)
N2—C3—C4—N1	−2.6 (12)	C2—C10—C11—C12	177.4 (6)
C9—N3—C5—C6	1.7 (9)	C10—C11—C12—C13	−0.1 (11)
Pt1—N3—C5—C6	−172.5 (5)	C11—C12—C13—C14	−0.3 (11)
C9—N3—C5—C1	−179.6 (6)	C12—C13—C14—N4	0.8 (11)
Pt1—N3—C5—C1	6.2 (8)	C10—N4—C14—C13	−0.8 (10)
N1—C1—C5—N3	−133.7 (6)	Pt1—N4—C14—C13	175.3 (5)
C2—C1—C5—N3	51.5 (9)		

### Hydrogen-bond geometry (Å, °)

**Table:**

*D*—H···*A*	*D*—H	H···*A*	*D*···*A*	*D*—H···*A*
C6—H6···Br1^i^	0.95	2.88	3.524 (6)	126.
C11—H11···Br1^ii^	0.95	2.88	3.688 (7)	143.

Symmetry codes: (i) *x*+1, *y*, *z*; (ii) −*x*+3/2, *y*+1/2, −*z*+1/2.
